# Recurrent infection transiently expands human tissue T cells while maintaining long-term homeostasis

**DOI:** 10.1084/jem.20210692

**Published:** 2023-06-14

**Authors:** Veronica Davé, Laura E. Richert-Spuhler, Tanvi Arkatkar, Lakshmi Warrier, Thepthara Pholsena, Christine Johnston, Joshua T. Schiffer, Martin Prlic, Jennifer M. Lund

**Affiliations:** 1 Vaccine and Infectious Disease Division, Fred Hutchinson Cancer Research Center, Seattle, WA, USA; 2 Department of Global Health, Graduate Program in Pathobiology, University of Washington, Seattle, WA, USA; 3 Department of Medicine, University of Washington, Seattle, WA, USA; 4 Clinical Research Division, Fred Hutchinson Cancer Research Center, Seattle, WA, USA; 5 Department of Immunology, University of Washington, Seattle, WA, USA

## Abstract

Chronic viral infections are known to lead to T cell exhaustion or dysfunction. However, it remains unclear if antigen exposure episodes from periodic viral reactivation, such as herpes simplex virus type-2 (HSV-2) recrudescence, are sufficient to induce T cell dysfunction, particularly in the context of a tissue-specific localized, rather than a systemic, infection. We designed and implemented a stringent clinical surveillance protocol to longitudinally track both viral shedding and in situ tissue immune responses in a cohort of HSV^+^ volunteers that agreed to avoid using anti-viral therapy for the course of this study. Comparing lesion to control skin biopsies, we found that tissue T cells expanded immediately after reactivation, and then returned numerically and phenotypically to steady state. T cell responses appeared to be driven at least in part by migration of circulating T cells to the infected tissue. Our data indicate that tissue T cells are stably maintained in response to HSV reactivation, resembling a series of acute recall responses.

## Teaser

The immune cell compartment in human genital skin expands and contracts following HSV-2 reactivation, and includes cells recruited from circulation and proliferating cells. Despite frequent antigen exposure, tissue T cell number and phenotype remain stable in the long-term, likely driven at least in part by cell-extrinsic and cell-intrinsic regulatory mechanisms.

## Graphical Abstract

**Figure:**
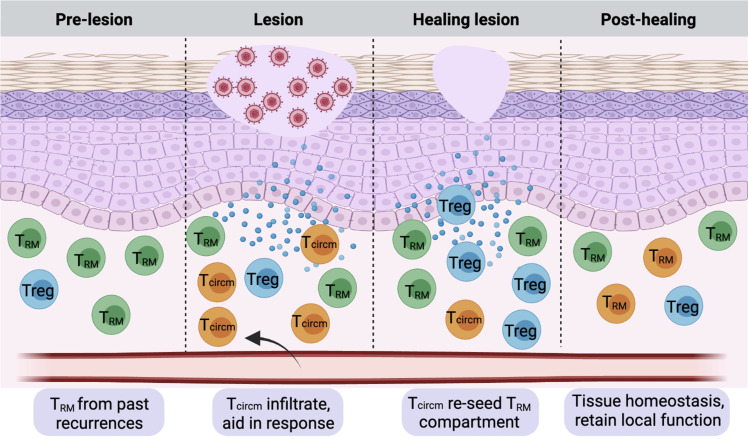

## Introduction

Herpes simplex virus type-2 (HSV-2) is a globally prevalent virus that establishes a lifelong infection characterized by the presence of episodic viral shedding and recrudescent genital ulcers. Resident memory T cells (T_RM_) are key contributors to the immune response to HSV infection (Shin and Iwasaki, 2013). Studies of human genital skin and mucosa have identified local clusters of antigen-specific CD8 and CD4 T_RM_ at sites of acute HSV-2 lesions and mucosa (Koelle et al., 2000; Peng et al., 2012; Posavad et al., 2017; Zhu et al., 2007; Zhu et al., 2009; Zhu et al., 2013). Together, data acquired from human and mouse studies demonstrate that T_RM_ abundantly congregate in response to HSV infection, and further, that they are necessary to achieve viral control (Gebhardt et al., 2009; Iijima and Iwasaki, 2014). However, little is known regarding their ability to retain functionality in the face of chronic antigen exposure, as occurs in repetitive human HSV-2 reactivation episodes (Schiffer and Corey, 2013), although the stability of an individual’s HSV-2 shedding rate over many years suggests globally stable antiviral immune function (Phipps et al., 2011).

Constant antigen exposure, such as that which occurs during systemic chronic infection and cancer, has been shown to divert the development of functional, self-renewing memory T cells into an exhausted phenotype (Tex; Wherry and Kurachi, 2015). Tex cells express inhibitory molecules such as PD-1, CTLA-4, Tim-3, CD244, LAG-3, CD101, and CD39, and exhibit impaired proliferative and survival potential, as well as impaired production of IL-2, TNF, and IFN-γ (Gupta et al., 2015; Hudson et al., 2019; Wherry and Kurachi, 2015). Studies of chronic systemic human infections such as HIV and hepatitis C virus, plus mouse studies using lymphocytic choriomeningitis virus clone 13, have contributed greatly to defining Tex phenotypes and identifying their loss of functionality (Trautmann et al., 2006; Urbani et al., 2006; Wherry et al., 2003). However, it remains unknown how chronic or episodic pathogen persistence in peripheral tissues, for example in the context of human HSV-2, may affect T cell fitness. Here we examined T cell responses within HSV-2 lesion sites over 24 wk, spanning early clinical presentation through healing to determine the fate, phenotype, and regulatory mechanisms governing T_RM_ populations facing periodic antigen exposure.

## Results and discussion

### Local T cell numbers expand upon HSV-2 lesion formation, but the overall composition of the immune cell compartment in tissue remains stable

**Figure 1.**
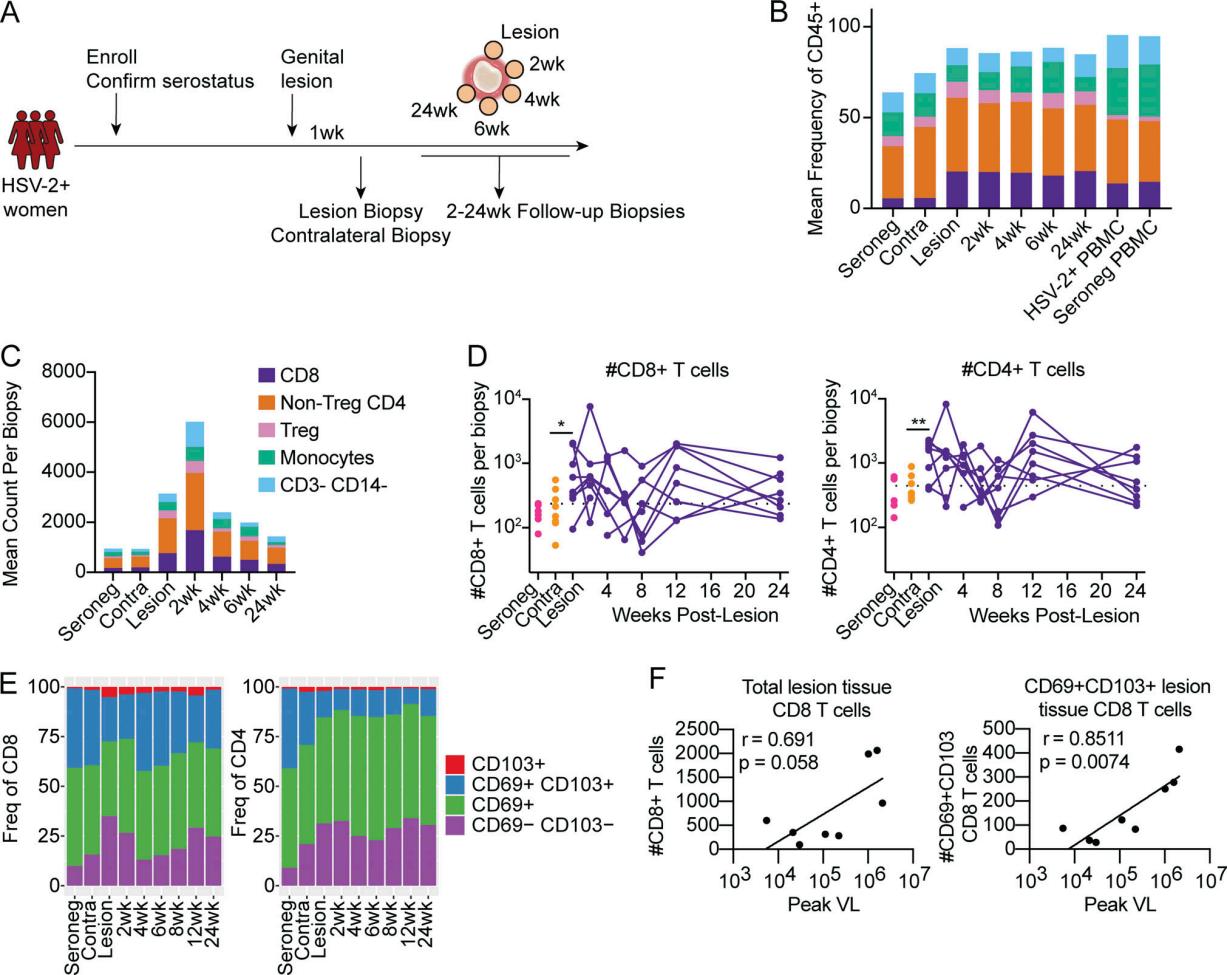
**The tissue T cell compartment expands and contracts with HSV-2 lesion formation and healing. (A)** Study schematic. **(B and C)** Mean frequencies (B) and total count (C) of indicated subsets of lymphocytes and CD14^+^ monocytes from genital biopsies or PBMCs as determined by flow cytometry. **(D)** Number of CD8 and CD4 T cells present in quiescent skin or healing lesion sites. The dotted line indicates mean number of cells in the contralateral biopsies. **(E)** Proportions of cells from quiescent skin or healing lesion sites co-expressing CD69 and CD103 over time. **(F)** Simple linear regression analysis of the peak HSV-2 viral load compared to the number of total CD8 T cells or CD69^+^CD103^+^ CD8 T cells present in the lesion biopsy. R and P values are from the Pearson correlation analysis. Best fit calculated by simple linear regression. *, P < 0.05; **, P < 0.01; calculated using paired *t* tests to compare contralateral skin to lesions at first collection. Data shown are from up to eight HSV-seropositive study participants, with samples collected longitudinally, and from six HSV-seronegative study participants.

**Figure S1.**
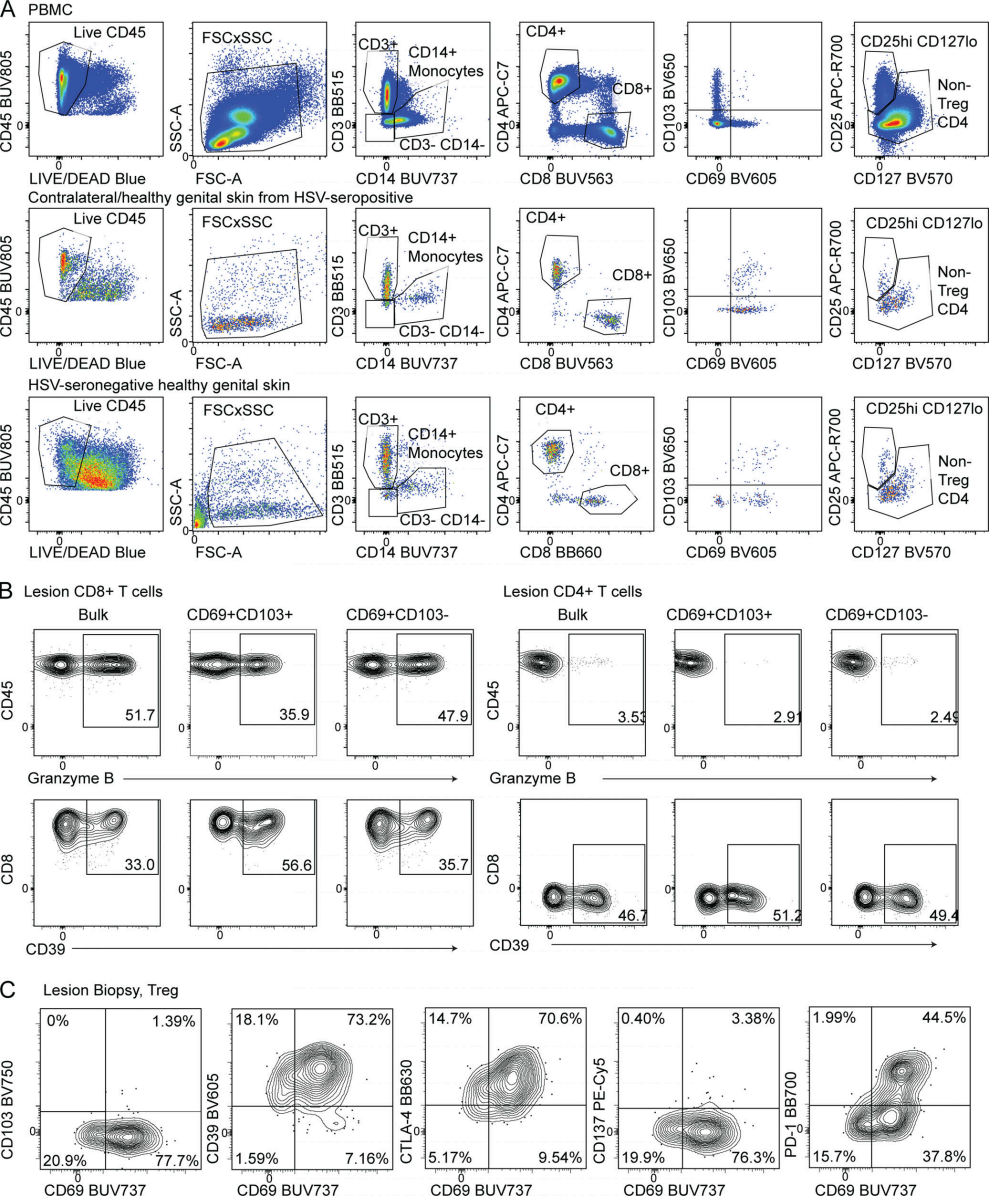
**Representative flow cytometry gating for human cells. (A)** Gating strategy to identify human CD4 T_RM_, CD8 T_RM_, and Treg populations. **(B)** Representative flow cytometry gating of granzyme B and CD39 among human CD69^+^ and CD69^+^CD103^+^ tissue CD8 or CD4 T cells. **(C)** Representative flow cytometry staining of human Tregs expressing markers of tissue residency and activation. Treg are defined as CD3^+^CD4^+^CD25^+^CD127^−^ cells.

We assessed the size and composition of the immune cell compartment at the site of an active HSV-2 lesion beginning as early as 1 wk after lesion presentation through 24 wk of healing. Immune cells were examined by collection of repeat lesion site biopsies in a daisy chain pattern (Fig. 1 A). In addition, we collected biopsies of unaffected contralateral skin from HSV-2–seropositive women 1 wk after they had experienced an HSV-2 lesion at a disparate site. Finally, as an additional control, site-matched genital skin was collected from seronegative participants. Using high-parameter flow cytometry, we determined the frequency and phenotype of T cells and monocytes present in HSV-2–seropositive skin over time versus seronegative skin (Fig. S1 A and Fig. 1, B–D). Peripheral blood mononuclear cells (PBMC) were run in parallel as an internal control and to aid in setting analysis gates. When we compared the composition of cells in contralateral skin to acute HSV-2 lesions, we identified a significant expansion of monocytes, CD8 and CD4 T cells, and Tregs in HSV-2 lesion sites (Fig. 1, B–D). The magnitude of T cell expansion was especially evident at early timepoints 1–4 wk after recurrence and was followed by an apparent contraction phase of tissue healing and a return to baseline (Fig. 1 D). We further characterized tissue T cells based on their expression of the tissue retention and residency markers, CD69 and CD103 (Sasson et al., 2020; Szabo et al., 2019), and found that CD69^+^CD103^−^ (single positive; SP) T cells made up the dominant proportion among total T cells, with CD69^+^CD103^+^ (double positive; DP) T cells contributing as well (Fig. 1 E). Finally, we sought to determine if the abundance of CD8 T cells in the lesion biopsy was related to the peak viral load of the HSV-2 reactivation episode and found that there a trend toward a positive correlation between the peak viral load and the number of CD8 T cells present in the lesion biopsy. Additionally, there was a positive correlation between the peak viral load and the number of CD69^+^CD103^+^ CD8 T_RM_ cells present in the lesion biopsy (Fig. 1 F), potentially indicating an expansion of T_RM_ cells related to severity of viral replication. Taken together, we found that over the course of acute HSV-2 lesion healing the composition of the immune cell compartment remained relatively unaltered, with all analyzed subsets exhibiting expansion and contraction of proportionate magnitude. Thus, whereas demonstrable expansion of tissue T cells occurred in response to HSV-2 lesion presentation, the overall composition of the tissue compartment remained similar to contralateral and HSV-2–seronegative genital skin during lesion healing.

### T cell expansion in HSV-2 lesions is due to a combination of in situ proliferation and recruitment from the periphery

**Figure 2.**
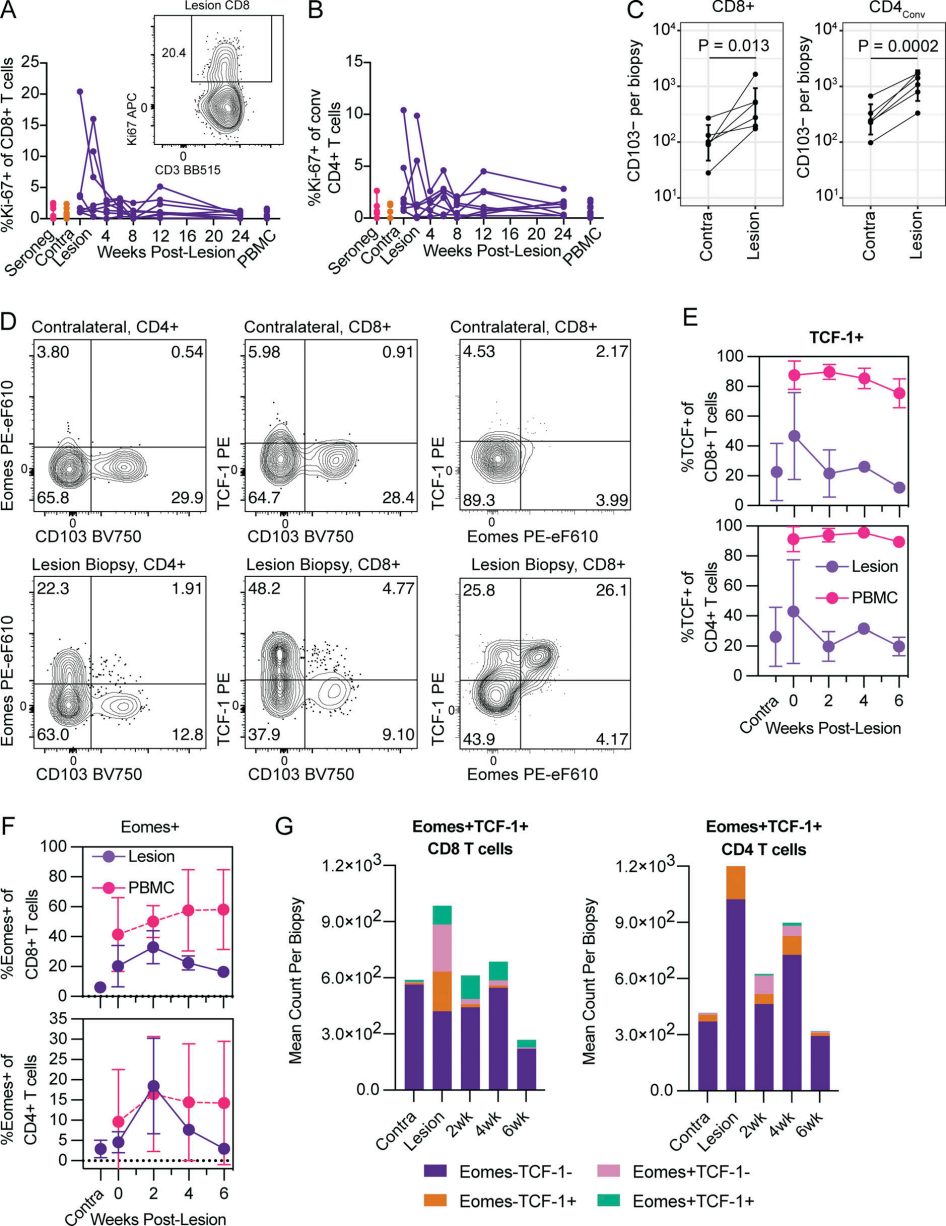
**Evidence of tissue T cell proliferation in lesion sites and T**_**RM**_ **precursors from the circulation seeding tissues during HSV-2 lesion healing. (A and B)** Representative flow cytometry plots demonstrating Ki-67 expression on CD8 tissue T cells, and frequencies of Ki-67^+^ CD8 or CD4 T cells in quiescent skin or over the course of lesion healing as compared to matched PBMCs. Data shown are from up to eight HSV-seropositive study participants, with samples collected longitudinally, and from six HSV-seronegative study participants. **(C)** CD103^−^ CD8 and CD4 T cells are more abundant in lesion sites compared to unaffected contralateral skin. **(D)** Representative flow cytometry plots demonstrating staining patterns of Eomes, TCF-1, and CD103 expression among tissue T cells from contralateral skin and lesion sites. **(E)** TCF-1 expression over 6 wk of lesion healing among tissue or peripheral CD8 and CD4 T cells. **(F)** Expression of Eomes among CD8 and CD4 T cells from tissue and PBMCs over 6 wk of lesion healing. **(G)** Distribution of absolute numbers of CD8 and CD4 T cells expressing Eomes and/or TCF-1 over 6 wk of lesion healing. Data shown in C–G are from three HSV-seropositive study participants, with samples collected longitudinally. P values were calculated using paired *t* tests where indicated.

T_RM_ are responsible for both immediate local antiviral activity, and aid the recruitment of recirculating memory T cells into the tissue through cytokine secretion (Beura et al., 2019; Jiang et al., 2012; Mackay et al., 2015; Schenkel et al., 2014; Schenkel et al., 2013). Furthermore, local proliferation of T_RM_ (which is independent from circulating memory T cell [Tcircm] recruitment) has been demonstrated and may be a mechanism to support durable T_RM_ immunity (Beura et al., 2018; Park et al., 2018). Our related work has corroborated these data and demonstrated that indeed, only small numbers of T_RM_ are required to eliminate virally infected cells (Roychoudhury et al., 2020). Based on these expectations, we sought to determine the magnitude of local T cell proliferation in HSV-2 lesion tissues as compared to recruitment of T cells from the blood. Using Ki-67 expression as a marker of recent proliferation, we compared tissue T cell rates in genital tissues from HSV-2 seronegative women, non-lesion contralateral skin, and healing HSV-2 lesions. We found heterogeneity in the frequency of tissue CD8 and CD4 T cell proliferation; both subsets of tissue T cells collected from the lesion site of some participants underwent robust early proliferation, returning to baseline frequencies by only 2 wk after lesion appearance for CD4 T cells and by 4 wk after lesion appearance for CD8 T cells (Fig. 2, A and B). At least three other participants demonstrated similar results although the kinetics of expansion and contraction were comparatively delayed. Ki-67 upregulation was less evident in the healing lesion tissue from the remaining participants, nor was Ki-67 expression correlated with the overall quantity of CD8 or CD4 T cells present in lesion sites (Pearson correlations; R^2^ = 0.363 and P = 0.114 for CD8 and R^2^ = 0.030 and P = 0.681 for CD4 T cells). Given the notable expansion of bulk T cells at the lesion site in all participants examined (Fig. 1), juxtaposed with negligible Ki-67 expression in many individuals, we hypothesized that T cell recruitment from the circulation must at least transiently contribute to the tissue compartment.

We next looked for evidence of potential T_RM_ precursor cell influx into the tissue. We first determined the magnitude of CD103 expression among cells present in contralateral versus lesion tissue and found that significantly more CD8 and CD4 T cells in lesion sites were CD103-negative (Fig. 2 C). These data suggest that CD103^−^ non-T_RM_ cells traffic to HSV-2 lesion sites, as has been recently suggested for intestinal tissue (Fung et al., 2022; von Hoesslin et al., 2022), and possibly represent a T_RM_ precursor derived from Tcircm cells (Beura et al., 2018; Park et al., 2018). Moreover, we expected such precursor tissue T cells to progressively downregulate the transcription factors TCF-1 and Eomes as they more stably acquired a T_RM_ phenotype over time (Davé et al., 2021; Mackay and Kallies, 2017). Indeed, we found that CD8 and CD4 T cells present in homeostatic tissue (contralateral biopsies) were Eomes^−^ and TCF-1^−^ (Fig. 2, D–G), whereas TCF-1^+^Eomes^+^ cells appeared during early lesion and healing (Fig. 2, D–G). Since effector T cells express low levels of TCF-1, these TCF-1^+^Eomes^+^ T cells could be Tcircm that are recruited to the tissue by inflammatory cues (Arkatkar et al., 2023; Maurice et al., 2019). Quantifying these results over the first 6 wk of lesion healing, we found that tissue T cells progressively downregulated TCF-1 expression, which resulted in TCF-1^−^ tissue T cells comprising the dominant population by week 6. These results were consistent with low TCF-1 expression by tissue T cells in quiescent contralateral skin, and in contrast to peripheral T cells which stably express TCF-1 (Fig. 2 E). Similarly, tissue T cells present in unaffected genital skin expressed minimal Eomes, whereas during lesion presentation we observed a robust early increase in Eomes^+^ CD8 and CD4 tissue T cell frequencies (Fig. 2 F). By 6 wk after acute lesion presentation, Eomes expression was minimal among tissue T cells, resembling the contralateral site. Overall expansion and contraction of the number of TCF-1 and Eomes expressing tissue T cells was similar to the trends observed for cell frequency (Fig. 2 G). Together these data suggest a return to baseline during 2–4 wk of lesion healing. We hypothesize that a subset of infiltrating Eomes^+^ Tcircm may establish residence in the tissue and subsequently downregulate Eomes, consistent with transcriptional profiles associated with stable T_RM_ populations (Behr et al., 2018; Crowl et al., 2022; Mackay and Kallies, 2017; Milner and Goldrath, 2018).

### Acute HSV-2 lesions elicit localized activation among both resident and infiltrating T cells

**Figure 3.**
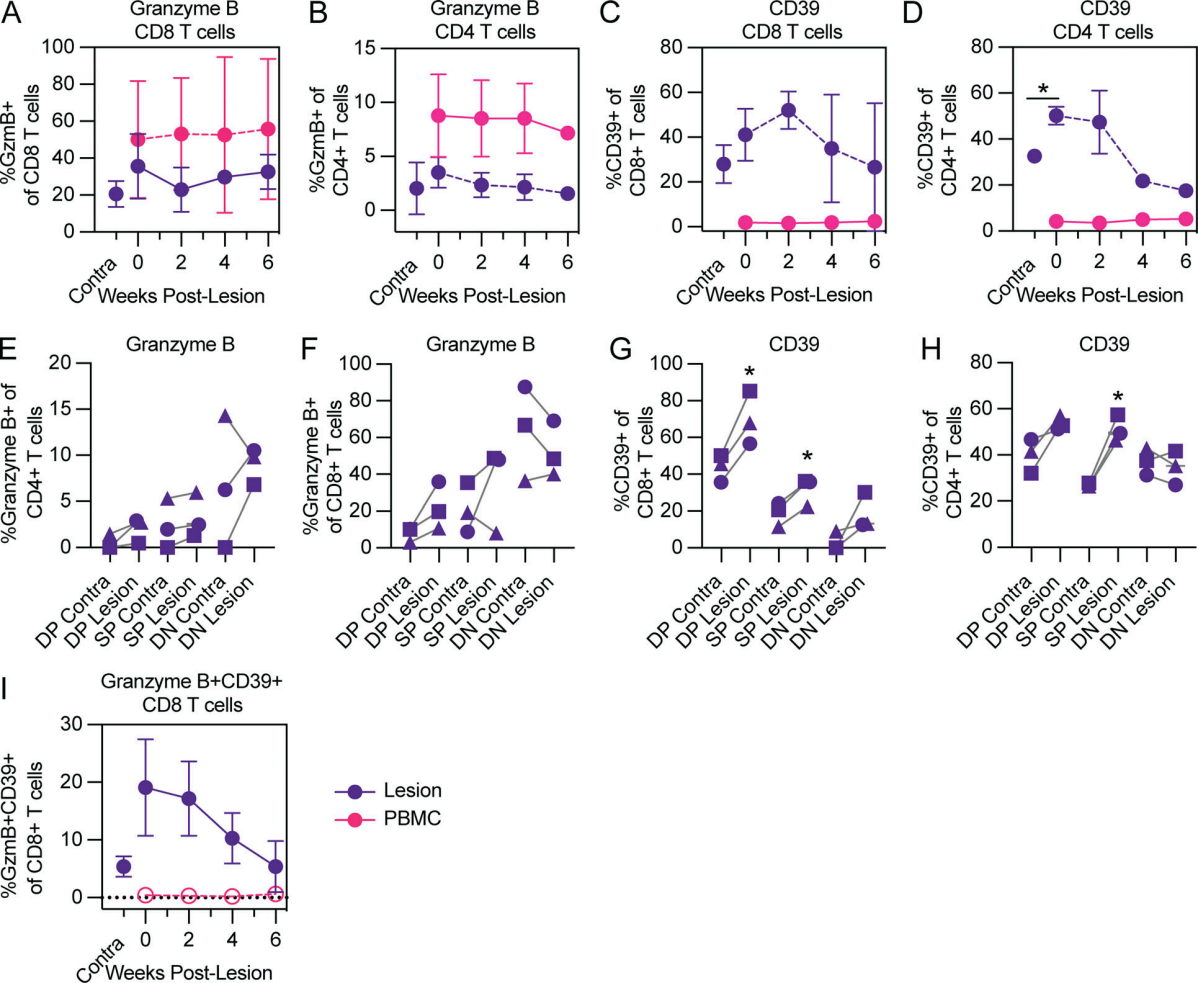
**Tissue T cells in HSV-2 lesions are transiently activated. (A–D and I)** Frequencies of granzyme B and CD39 expression by bulk tissue CD8 and CD4 T cells or PBMCs over the course of lesion healing. **(E–H)** Expression of granzyme B and CD39 among CD69^−^CD103^−^ doubly negative (DN), CD69^+^CD103^−^ SP, or CD69^+^C103^+^ DP tissue CD8 and CD4 T cells at the lesion timepoint. *, P < 0.05; paired *t* tests were used to compare contralateral tissue to lesion sites. Data shown are from three HSV-seropositive study participants, with samples collected longitudinally.

We next assessed the activation and functional specialization profiles of bulk tissue T cells or CD69^+^CD103^+^ (DP) or CD69^+^CD103^−^ (SP) T_RM_ cells. Comparing participant-matched contralateral skin to healing lesion sites, we found that lesion CD8 and CD4 T cells expressed activation or functional specialization markers, likely indicative of their antiviral capacities (Fig. 3). Bulk CD8 and CD4 T cells expressed more granzyme B in lesion tissue than in contralateral tissue, but this increase did not reach statistical significance, possibly due to the limited number of participants examined, which was in part due to the intensive nature of the longitudinal sampling, as well as clinic constraints due to COVID-19. Conversely, granzyme B expression among peripheral blood T cells was stable throughout the collection period (Fig. S1 B and Fig. 3, A and B). The percentage of bulk CD8 and CD4 T cells expressing the activation marker CD39 was significantly increased among CD4 T cells within the first 1–2 wk after lesion presentation (Fig. S1 B and Fig. 3, C and D). After week 2 post-lesion, the spike in the frequency of bulk T cells responding to viral insult tapered off in an apparent return to baseline, consistent with our hypothesis that following an episode of HSV-2 recurrence, tissue T cell function returns to steady state or quiescent levels. We also explored whether granzyme B or CD39 expressing tissue T cells were CD69^+^ and CD103^+^. Consistent with our expectations for the lesion site, frequencies of activated CD69^+^CD103^+^ DP and CD69^+^CD103^−^ SP tissue CD8 T cells were increased in most cases as compared to contralateral skin (Fig. 3, E–H). These results were particularly clear for activated tissue CD8 T cells expressing CD39 (Fig. 3 G). Within the CD4 T cell compartment we found minimal differences amongst granzyme B expressing tissue T cells, although the proportion of CD39 expressing SP cells was significantly higher at lesion sites (Fig. 3 H).

Finally, we evaluated the possibility for circulating T cells to seed lesion tissue (Beura et al., 2018; Park et al., 2018). We measured CD8 T cells co-expressing granzyme B, CD39, Ki-67, and TCF-1 as a proxy for Tcircm infiltrating HSV-2 lesion tissue. However, as demonstrated in Fig. 2, TCF-1^+^ cells rapidly decrease in frequency upon tissue entry into HSV-2 lesion sites and Ki-67 expression was variable among donors, leading us to focus on CD8^+^granzyme B^+^CD39^+^ tissue T cells. Consistent with observed patterns of SP granzyme B or CD39 tissue T cells, granzyme B^+^CD39^+^ tissue T cells infiltrated tissue early after lesion formation and decreased over the course of lesion healing to levels akin to quiescent skin, whereas CD8^+^granzyme B^+^CD39^+^ T cells among PBMCs were stably low (Fig. 3 I).

### Chronically recurrent antigen exposure does not appear to result in T_RM_ exhaustion

**Figure 4.**
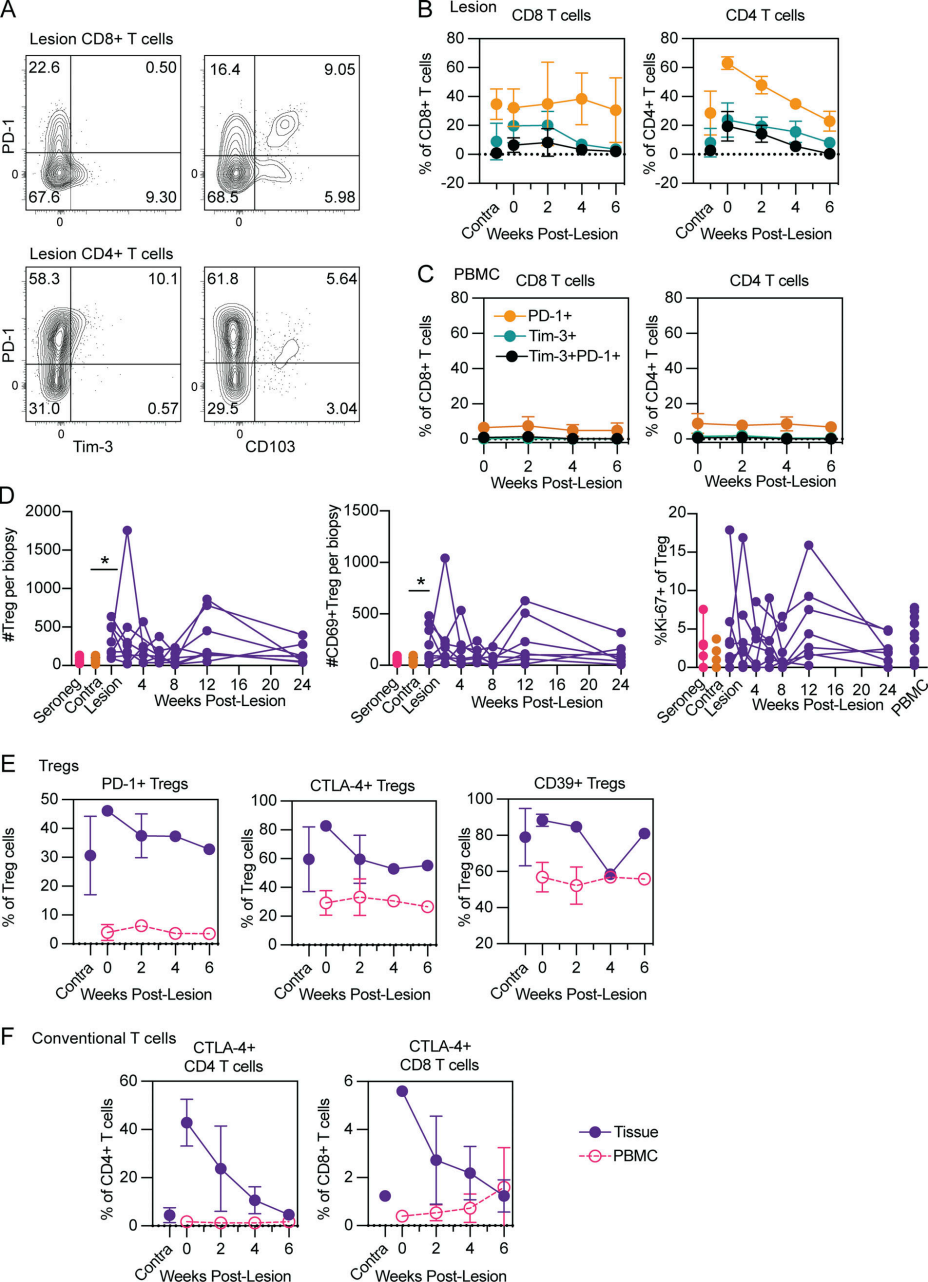
**Tissue T cells do not acquire an exhausted phenotype, and T cell–intrinsic and –extrinsic immunoregulatory mechanisms may contribute to maintenance of T cell resilience. (A)** Representative flow cytometry staining for CD8 and CD4 T cells expressing markers of exhaustion and tissue residency. **(B and C)** Frequencies of tissue (B) or circulating (C) CD8 and CD4 T cells expressing PD-1, Tim-3, or both markers over 6 wk of lesion healing compared to contralateral skin. Data shown in B and C are from three HSV-seropositive study participants, with samples collected longitudinally. **(D)** Absolute number of Tregs, and those expressing CD69 (left and center) or frequencies or Ki-67^+^ Tregs as compared to quiescent skin and PBMCs (right). Treg are defined as CD3^+^CD4^+^CD25^+^CD127^−^ cells. Data shown are from up to eight HSV-seropositive study participants, with samples collected longitudinally. **(E)** Frequencies of Treg activation markers as compared to matched PBMCs over 6 wk of lesion healing. **(F)** Expression of CTLA-4 on conventional CD4 and CD8 T cells compared to PBMCs over 6 wk of lesion healing. Data shown in E and F are from three HSV-seropositive study participants, with samples collected longitudinally. *, P < 0.05; paired *t* tests were used to compare contralateral tissue to lesions at the first collection.

Given our observation that T cells present in HSV-2 lesion sites express multiple activation markers during the early weeks following a lesion, we performed an exploratory analysis on a small subset of patients to determine if recurrent and frequent exposure to viral antigens and a heightened state of activation may lead to T cell exhaustion. We assessed staining patterns for several combinations of CD8 T cell exhaustion markers including PD-1 and Tim-3 (Fig. 4 A). Over the course of lesion healing we observed stable PD-1 and Tim-3 expression by CD8 T cells, similar to levels in contralateral skin, whereas CD4 T cells are characterized by an early peak of activated cells, followed by a decline to baseline frequencies akin to contralateral skin (Fig. 4 B). When we considered co-expression of PD-1 and Tim-3 as a more stringent indication of T cell exhaustion phenotypes, we found minimal frequencies of PD-1^+^Tim-3^+^ CD8 and CD4 T cells in the lesion and throughout healing (Fig. 4, A and B). Among PBMCs we found only minimal expression of exhaustion markers, which remained stable over time, as might be expected in response to a localized infection (Fig. 4 C). In this regard, although we found transiently increased frequencies of PD-1^+^ and Tim-3^+^ cells among tissue T cells at lesion sites, transient display of PD-1 and Tim-3 is not sufficient to determine Tex acquisition. Therefore, although the genital skin of HSV-2 seropositive women experiences chronic exposure to viral antigen, upon lesion healing, tissue T cells do not acquire phenotypes associated with chronic exhaustion.

### T cell–intrinsic and –extrinsic immunoregulatory mechanisms coordinate T_RM_ fitness

We next sought to identify potential regulatory mechanisms governing the observed T cell fitness, as opposed to exhausted phenotypes in lesion tissues. Regulatory T cells (Treg) utilize diverse mechanisms of immunomodulation to both promote effector responses during acute infection, and limit tissue pathology (Lund et al., 2008; Richert-Spuhler and Lund, 2015; Traxinger et al., 2022). Furthermore, Tregs have been identified by microscopy to be abundantly present in the human HSV-2 lesion site (Milman et al., 2016). Thus, we assessed the overall counts, frequencies, and phenotype of Tregs present in healing lesion tissue over time as compared to tissue Tregs present in quiescent genital skin. Bulk Tregs and those expressing CD69 were significantly more abundant in lesion tissues during the first 2 wk after lesion presentation, followed by an apparent return to homeostatic frequencies after week 2 (Fig. S1 C and Fig. 4 D). The frequency of tissue Tregs expressing Ki-67 was also elevated in healing lesion tissue, although amongst individual women, the timing of Ki-67 responses were heterogenous (Fig. 4 D). We also compared the tissue compartment to activation patterns of circulating Tregs from matched PBMCs and found significantly upregulated expression of PD-1, CTLA-4, and CD39, especially early after lesion presentation and healing (Fig. 4 E). Tregs modulate immune responses through a variety of mechanisms. For example, PD-1 promotes the survival and suppressive capabilities of Tregs (Asano et al., 2017); the ectoenzyme CD39 coordinates with CD73 (also expressed on Tregs) to hydrolyze ATP to immunosuppressive adenosine (Deaglio et al., 2007); and CTLA-4 competitively binds B7 molecules to inhibit co-stimulatory activity (Josefowicz et al., 2012). We found that expression of PD-1, CTLA-4, and CD39 on tissue Tregs followed similar kinetics as we had observed for other T cell populations, namely an early upregulation, as compared to contralateral skin, followed by the return to baseline upon lesion healing (Fig. 4 E). Moreover, consistent with the localized nature of HSV-2 viral shedding, the frequencies of activated tissue Tregs were dynamic over the course of lesion presentation and healing, while the frequencies of circulating Tregs expressing the same activation markers among PBMCs were stable. Finally, we evaluated expression of CTLA-4 on conventional CD4 and CD8 T cells. Consistent with our T cell activation data, CTLA-4 expression was dramatically increased in lesion tissue and returned to levels akin to quiescent skin by week 6 of lesion healing. Meanwhile, frequencies of CD4 and CD8 T cells among PBMCs expressing CTLA-4 were stably low (Fig. 4 F). Together, our data indicate that tissue Tregs become activated in response to HSV-2 lesion presentation; and whereas tissue Tregs temporally adjust their activation patterns concordant with tissue healing, peripheral Tregs maintain a phenotype distinct from tissue Treg over time. Therefore, we propose that both Tregs and T cell–intrinsic immunoregulatory mechanisms including CTLA-4 signaling assist in maintaining T_RM_ fitness in the context of HSV-2 reactivations and lesion healing.

### HSV-specific and circulating CD8 T cells predominate the early tissue response to recurrent localized infection

**Figure 5.**
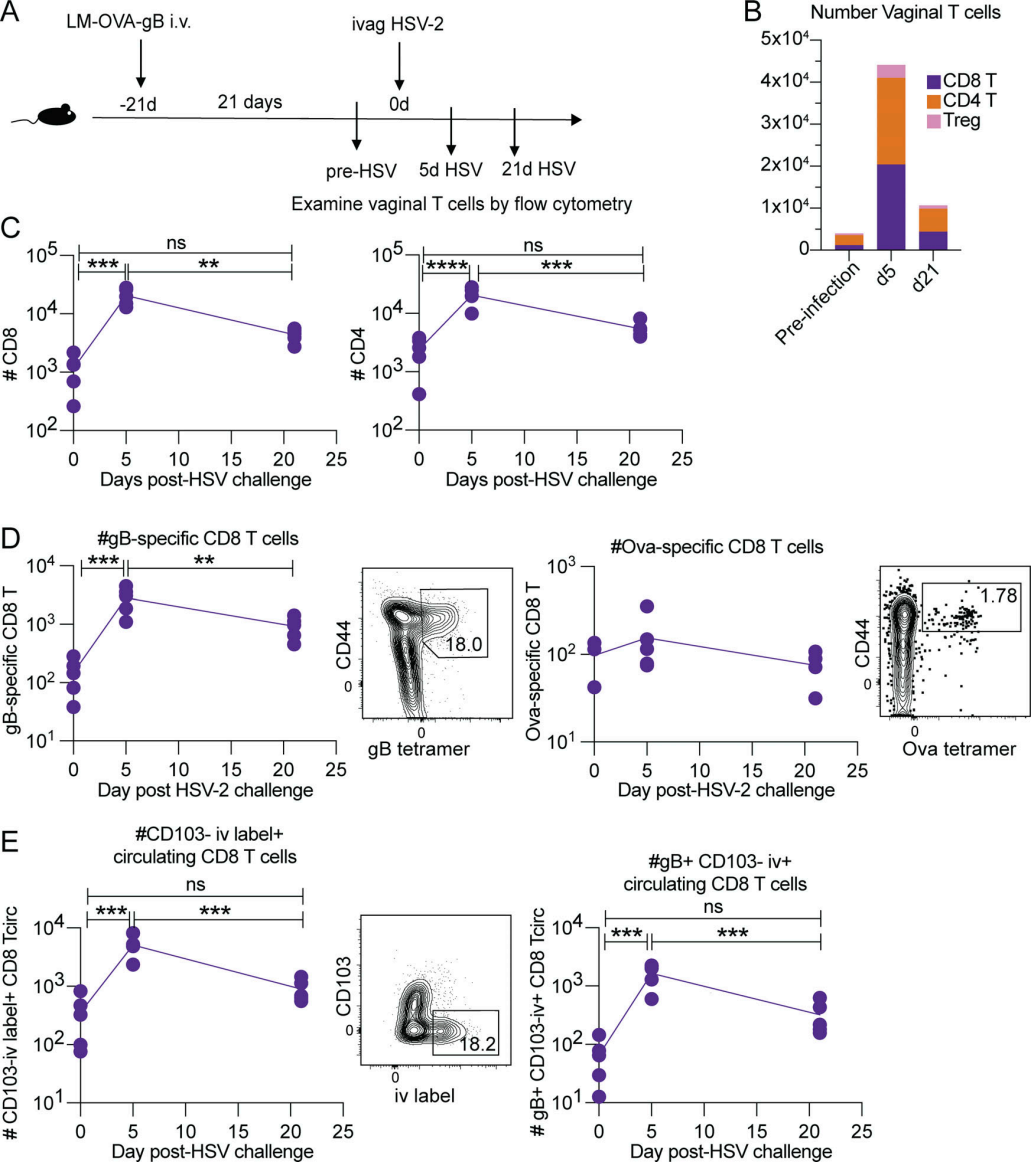
**HSV-specific and recently circulating T cells predominate the tissue T cell response during recurrent localized infection. (A)** Mouse experiment schematic. Mice were infected i.v. with LM-OVA-gB, and then challenged vaginally with an attenuated Tk^−^ strain of HSV-2. Cohorts of mice were euthanized 21 d after LM-OVA-gB infection (“pre-HSV infection”), 5 d after HSV-2 challenge, and 21 d after HSV-2 challenge. **(B)** Mean count of indicated subsets of lymphocytes in the vaginal tract as determined by flow cytometry. **(C and D)** Number of CD8 and CD4 T cells present in mouse vaginal tract ranging from days 0–21 after HSV-2 infection. CD8 T cells in the vaginal tract were further assessed for gB- and OVA-tetramer–positive subsets (D). **(E)** Just prior to euthanasia, mice received i.v. injection with anti-CD8 antibody (intravascular staining). Bulk CD8 T cell or gB-specific CD8 T cell populations were then categorized as circulating T cells based on being i.v. label–positive and CD103-negative. *, P < 0.05; **, P < 0.01; ***, P < 0.001; ****, P < 0.0001; calculated using one-way ANOVA with Tukey’s multiple comparisons test. Data shown are representative of two experiments, and five mice were included in each group/timepoint.

Based on our human data, we propose that recruitment of peripheral T cells to the lesion occurs following an HSV reactivation episode. To directly assess if antigen non-specific memory T cells are recruited to the site of HSV infection, we next utilized a mouse model system. Mice were infected with LM-OVA-glycoprotein B (gB) to seed CD8 T cells specific for HSV as well as for an antigen irrelevant to HSV-2 infection, OVA, such that we could track both populations following a vaginal infection with HSV-2 (Fig. 5 A). We have previously demonstrated that i.v. infection with LM-OVA-gB results in a population of tissue-resident memory CD8 T cells (Davé et al., 2021), thereby allowing us to examine the phenotype and function of memory CD8 T cells in the genital tissue of mice after a vaginal challenge with HSV-2. Therefore, we examined vaginal T cells in mice prior to HSV-2 infection (pre-infection), which we consider to be similar to the contralateral biopsy from our human cohort, as well as at days 5 and 21 after HSV-2 infection. Similar to our findings in human genital skin (Fig. 1, C and D), we observed a significant surge in the T cell population at an early timepoint after a T cell recall response to HSV, and counts returned to baseline by day 21 after infection (Fig. 5, B and C). Further, we found that both the HSV-specific and OVA-specific populations of CD8 T cells increased in number upon HSV-2 re-infection, followed by a contraction back to baseline levels (Fig. 5 D). Based on these findings, we conclude that CD8 T cell numbers return to steady state following the acute infection stage of an infection episode in both mouse and humans rather than leading to an erosion of HSV non-specific CD8 T cells at the infection site over the long-term. We hypothesize that this could serve to maintain barrier immunity to a spectrum of pathogens.

We also used the mouse model to address the origin of the increase in CD8 T cell number at the site of recurrent local infection using both intravascular staining (Galkina et al., 2005) as well as the tissue-resident marker CD103. Importantly, the mouse studies allow for enumeration of cells within the vasculature of tissues rather than within the tissue parenchyma. Similar to our data from HSV-infected humans (Fig. 2), we observed that circulating CD8 T cells were major contributors to the response early after an HSV-2 recurrence; specifically, we observed a significant increase in the number of CD103-negative, i.v. label–positive bulk CD8 T cells as well as gB-specific CD8 T cells 5 d after HSV-2 challenge as compared to the pre-infection timepoint (Fig. 5 E). Further, we observed a significant decrease in the number of these circulating CD8 T cells at 21 d after HSV-2 challenge, thereby indicating that either these circulating cells entered the tissue and converted into tissue-resident T cells, or they die or exit the tissue upon resolution of infection. Overall, this indicates that circulating T cells traffic to the site of localized recurrent infection to aid in viral clearance, and that the surge in T cell numbers found early after infection is not solely due to T_RM_.

**Figure S2.**
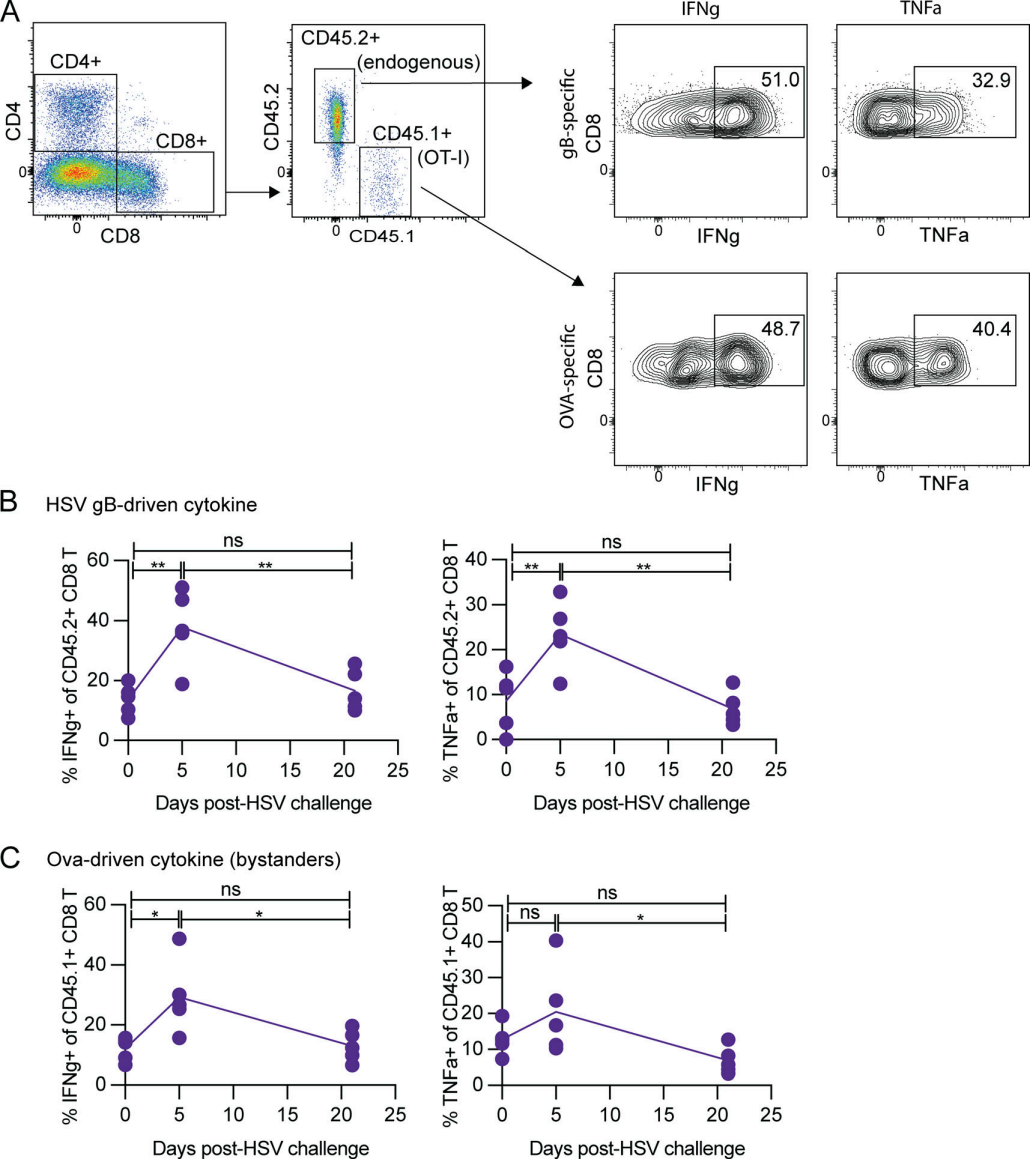
**Tissue CD8 T cells retain function upon recurrent localized infection.** 1 d prior to Listeria infection, B6 mice received adoptive transfer of 5,000 OT-I CD45.1 cells. B6, CD45.2 mice were then infected with LM-OVA-gB and Tk^−^ HSV-2, as in Fig. 5. At the indicated timepoints, vaginal cells were collected, stimulated ex vivo with gB and OVA peptide, and stained for intracellular cytokines by flow cytometry. **(A)** Representative staining from vaginal cells at day 5 after infection with HSV-2. **(B)** IFNγ and TNFα expression by CD45.2^+^ CD8 T cells (“HSV gB-driven cytokine”). **(C)** IFNγ and TNFα expression by CD45.1^+^ CD8 T cells (“Ova-driven cytokine”). *, P < 0.05; **, P < 0.01; ***, P < 0.001; ****, P < 0.0001; calculated using one-way ANOVA with Tukey’s multiple comparisons test. Data shown are representative of two experiments, and five mice were included in each group/timepoint.

In summary, data from human longitudinal skin biopsies and our mouse model is in line with the notion that an influx of memory T cells from the circulation supplements the tissue memory T cell response upon localized episodic infection. Such peripheral recruitment of memory T cells to a tissue site with activated T_RM_ has been demonstrated in mouse models (Fung et al., 2022; von Hoesslin et al., 2022), and further, circulating memory CD8 T cells that have been exposed several times to antigen have a heightened propensity to migrate to the lung and convert to T_RM_ upon influenza virus infection (Van Braeckel-Budimir et al., 2018). Observations from our human biopsy study and mouse experiments with HSV-2 infection of the genital tract reported herein are in line with these previously published findings and point to a potential role for T cells entering the tissue from the circulation during an infection episode to re-seed the T_RM_ population in order to maintain barrier protection. Supportive of this hypothesis, mathematical modeling indicates that this recruitment is a key method of control in human HSV-2 lesions (Roychoudhury et al., 2020). An important limitation of this result is that we did not measure T cell activity during the first crucial few days of HSV-2 reactivation and our modeling has suggested that proliferation of local T_RM_ is vital during this stage of infection (Schiffer et al., 2010; Schiffer et al., 2013). Furthermore, it is possible that rather than re-seeding the T_RM_ compartment, circulating T cells entering the tissue die off upon resolution of the infection episode, or exit the tissue rather than converting into new T_RM_. Furthermore, given the limitations of working with small human tissue biopsies, we were unable to examine the functional capacity of human tissue T cells to truly establish maintained fitness. Thus, we also employed a mouse model of recurrent exposure to HSV-2 to determine if tissue T cells retain functionality upon and subsequent to an infection episode. We found that tissue T cells retained their ability to express both IFNγ and TNFα after an HSV-2 infection episode (Fig. S2), supporting the notion that episodic tissue infection does not result in functional T cell exhaustion (Mackay et al., 2012). Overall, we demonstrate that the T cell response to a recurrent localized infection more closely resembles a series of acute recall response rather than development of an exhausted phenotype T cell response that occurs in settings of consistently chronic infection.

## Materials and methods

### Study population

Nine HSV-2–seropositive, HIV-seronegative, non-pregnant women with a history of symptomatic genital herpes with three or more outbreaks in the past year were enrolled into the study. Participants agreed to avoid the use of anti-HSV therapy during the study. Six HSV-2–seronegative women were also enrolled as controls. The median age of participants was 51, and participants had a median of four genital herpes recurrences/year. While awaiting a genital lesion recurrence, participants collected swabs twice daily and completed a daily diary. At the time of a recurrence, swabs were self-collected from the lesion site every 3 h during the day and once overnight for 6 d.

Genital biopsies at the lesion site were collected ∼1 wk after initial ulcer appearance to avoid collecting a biopsy from an actively ulcerative site. Each 3 mm punch biopsy of an acute lesion included 50% of the vesicle area and 50% of the immediately adjacent erythematous skin area. Genital biopsies were then collected at 2, 4, 6, 8, 12, and 24 wk after healing. Biopsies collected after lesion healing were collected in a daisy chain fashion from the predominant area of the lesion, usually contiguous to the prior biopsy. Control skin biopsies were collected from the opposite (contralateral) anatomic site of the HSV reactivation, as described elsewhere (Zhu et al., 2009; Zhu et al., 2007). We also enrolled six HSV-1– and HSV-2–seronegative participants who underwent a single labia majora or perineum biopsy (see list below). The protocol, biopsy procedures, and informed consent were approved by the University of Washington Institutional Review Board. All participants provided informed consent at the time of enrollment.

A summary of the study participants, including their biopsy location, weeks between ulcer appearance and first biopsy, HSV status, and which flow cytometry panel was run, is listed below.

Participant 1: Biopsied labia majora, 0.71 wk, HSV-2^+^, immune cell subset panel
Participant 2: Biopsied labia majora, 1.57 wk, HSV-2^+^, immune cell subset panel
Participant 3: Biopsied labia majora, 1.71 wk, HSV-2^+^, immune cell subset panel
Participant 4: Biopsied perineum, 2.57 wk, HSV-2^+^, immune cell subset panel
Participant 5: Biopsied perineum, 1.71 wk, HSV-2^+^, immune cell subset panel
Participant 5: Biopsied labia majora, 1 wk, HSV-2^+^, T cell activation/exhaustion panel
Participant 6: Biopsied mons, 1.86 wk, HSV-2^+^, immune cell subset panel
Participant 7: Biopsied perineum, 1.14 wk, HSV-2^+^, immune cell subset panel
Participant 8: Biopsied perineum, 1 wk, HSV-2^+^, T cell activation/exhaustion panel
Participant 9: Biopsied buttock, 1 wk, HSV-2^+^, T cell activation/exhaustion panel
Participant 10: Biopsied labia majora, HSV-seronegative, immune cell subset panel
Participant 11: Biopsied labia majora, HSV-seronegative, immune cell subset panel
Participant 12: Biopsied perineum, HSV-seronegative, immune cell subset panel
Participant 13: Biopsied labia majora, HSV-seronegative, immune cell subset panel
Participant 14: Biopsied perineum, HSV-seronegative, immune cell subset panel
Participant 15: Biopsied perineum, HSV-seronegative, immune cell subset panel

### Tissue collection and processing

Blood was collected into acid citrate dextrose tubes. Biopsies were transported from the clinic in ice cold unsupplemented Roswell Park Memorial Institute 1640 (RPMI) medium (Gibco) and immediately processed upon arrival. Biopsies were minced into pieces and subjected to two rounds of digestion in 10 ml RPMI supplemented with 2% FBS, 2 mg/ml collagenase D (Roche), and 1.5 mg/ml DNase I (Roche) for 30 min at 37°C with gentle rocking. Single-cell suspensions were washed and filtered before staining for flow cytometry.

### Cell staining for flow cytometry

Cells were first incubated in LIVE/DEAD Blue fixable amine-reactive viability dye (Thermo Fisher Scientific) for 10–20 min at room temperature. Two different flow panels were used to characterize immune cells. For the immune cell subset panel (see list below), CCR7 staining was performed at 37°C and all other staining was performed on ice. For the T cell activation and exhaustion panel (see list below), samples were blocked for Fc binding using Human TruStain (BioLegend), and then stained with antibodies on ice. Cytosolic and nuclear proteins were detected using Foxp3/Transcription Factor Fixation/Permeabilization reagents (eBioscience). Samples were acquired on a FACSymphony instrument (BD). Cell counts were determined by acquiring the full sample and determining the number of cellular events recovered.

#### Human immune cell subset panel

CD38-BUV395, clone HB7, BD
Viability, LIVE/DEAD blue, Thermo Fisher Scientific
CD8-BUV563, clone RPA-T8, BD
CD11c-BUV661, clone B-ly6, BD
CD14-BUV737, clone M5E2, BD
CD45-BUV806, clone HI30, BD
Helios-eFluor450, clone 22F6, eBioscience
CCR7-BV510, clone G043H7, BioLegend
CD127-BV570, clone A01905, BioLegend
CD69-BV605, clone FN50, BioLegend
CD103-BV650, clone Ber-ACTG8, BD
HLA-DR-BV711, clone L243, BioLegend
CD141-BV785, clone M80, BioLegend
CD3-BB515, clone HIT3a, BD
CD1c-BB700, clone F10/21A3, BD
TCF-1-PE, clone C63D9, Cell Signaling
Foxp3-PE-Dazzle594, clone 206D, BioLegend
CD123-PE-C75, clone 9F5, BD
DC-SIGN-PE-Cy7, clone 9E9A8, BioLegend
Ki67-APC, BD
CD25-APC-R700, clone 2A3, BD
CD4-APC-Cy7, clone OKT4, BioLegend

#### Human T cell activation and exhaustion panel

CD8-BUV395, clone RPA-T8, BD
Viability, LIVE/DEAD blue, Thermo Fisher Scientific
CD3-BUV496, clone UCHT1, BD
CD25-BUV563, clone 2A3, BD
CD69-BUV737, clone FN50, BD
CD45-BUV805, clone HI30, BD
Helios-eFluor450, clone 22F6, eBioscience
CCR7-BV510, clone G043H7, BioLegend
CD45RA-BV570, clone HI100, BioLegend
CD39-BV605, clone A1, BioLegend
Tim3-BV650, clone 7D3, BD
T-bet-BV711, clone 4B10, BioLegend
CD103-BV750, clone Ber-ACT8, BD
Ki67-BV785, clone B56, BD
Foxp3-AlexaFluor488, clone 259D/C7, BD
CTLA-4-BB630, clone BNI3, BD
CD127-Biotin/SA-BB660, clone A019D5, BioLegend
PD-1-BB700, clone EH12.1, BD
TCF-1-PE, clone C63D9, Cell Signaling
Eomes-PE-eFluor610, clone WD1928, eBioscience
CD137-PE-Cy5, clone 4B4-1, BD
CD19-PE-Cy5.5, clone MHCD1918, Invitrogen
IRF4-PE-Cy7, clone IRF4.3E4, BioLegend
Tox-APC, clone REA473, Miltenyi
Granzyme B-AlexaFluor700, clone GB11, BD
CD4-APH-H7, clone RPA-T4, BD

### Mouse experiments

6-wk-old female C57BL6/J mice were purchased from the Jackson Laboratory (strain #000664) and maintained in specific pathogen–free conditions at the Fred Hutchinson Cancer Research Center (Fred Hutch). Experiments were approved by the Fred Hutch Institutional Animal Care and Use Committee. To generate LM-OVA-gB memory mice, mice received i.v. immunization with *Listeria monocytogenes* strains that were generated to recombinantly express OVA with HSV-2 gB–derived peptide SSIEFARL according to previously described methods (Davé et al., 2021). The pPL2 vector was used to achieve integration into the bacterial genome. The mice received 4000 CFU of LM-OVA-gB and were rested for 21 d after immunization before they were infected with attenuated HSV (HSV Tk^−^). Mice were s.c. injected with 2 mg of medroxyprogesterone acetate injectable suspension (Depo-Provera) dissolved in sterile PBS 5–7 d before vaginal infection. For *HSV Tk*^*−*^ infection, mice were infected intravaginally with 1.88 × 10^7^ PFU of attenuated HSV-strain (Jones et al., 2000). For in vivo T cell labeling, CD8b monoclonal antibody (clone: H35-17.2, cat#11-0083-82; Thermo Fisher Scientific Scientific, FITC) was administered via intravascular route by retro-orbital injection 5 min before the mice were euthanized.

The vaginal tract and cervix were harvested and minced thoroughly in prewarmed digestion media which was freshly prepared and consisted of DMEM, collagenase D (Sigma-Aldrich, at 2 mg/ml), 500 μl dnase (15 mg/ml), and 10% FBS. The minced tissue was incubated for 30 min at 37°C on a shaker. Following incubation, the minced tissue was spun at 1,500 rpm for 5 min. To prepare single cell suspensions, the mixture was mashed through a 70 μm strainer. The flow panels that were used to characterize the mouse immune cells are listed below.

#### Mouse T cell phenotyping panel

CD8a-UV395, clone 53-6.7, BD
Viability, LIVE/DEAD blue, Thermo Fisher Scientific
CD62L-BUV737, clone MEL14, BioLegend
CD44-BUV661, clone IM7, BioLegend
Granzyme B-PacBlue, clone GB11, Thermo Fisher Scientific
Foxp3-eFluor506, clone FJK-16s, Thermo Fisher Scientific
CD013-BV786, clone BD M290, BD
Ki67-BV605, clone 16A8, BioLegend
PD-1-BV711, clone EH12.1, BioLegend
CD45-BV650, clone 30-F11
CD8b-FITC, clone eBioH35-17.2, Thermo Fisher Scientific
Tim3-PerCP5.5, clone 5D12, BD
OVA Kb tetramer-PE, Fred Hutch Core
CD69-PE TexasRed, clone H1.2F3, BioLegend
TCF-1-PE-Cy7, clone C63D9, BioLegend
gB tetramer-APC, Fred Hutch Core
Eomes-AlexaFluor700, clone 1219A, R&D
CD4-APC Cy7, clone GK1.5, Thermo Fisher Scientific

To detect cytokine production by CD8 T cells, intracellular cytokine staining was performed. For these experiments, 1 d prior to Listeria infection, B6 mice received adoptive transfer of 5,000 OT-I CD45.1 cells. B6, CD45.2 mice were then infected with LM-OVA-gB and Tk^−^ HSV-2 as described above. At various times after infection, single-cell suspensions were obtained as described above. Cells were plated in a 96-well plate and spun down at 1,500 rpm for 5 min. The supernatant was carefully removed and RP10 media containing a combination of peptides with Golgi Plug (1:1,000) was added: gB (HSV) peptide (10^8^ M) and Ova SIINFEKL peptide (200 nM) for 4 h.

#### Mouse intracellular cytokine staining panel

CD45.1-UV395, BD
Viability, LIVE/DEAD Blue, Thermo Fisher Scientific
IFNg-BUV737, clone XMG1.2, BD
Granzyme B-PacBlue, clone GB11, Thermo Fisher Scientific
CD103-BV786, clone BD M290, BD
PD-1-BV605, clone 29F.1A12, BioLegend
CD45-BV650, clone 30-F11, BD
CD8-BV711, clone 53-6.7, BioLegend
TNFa-PerCP, clone MP6-XT22, BioLegend
CD44-PE, clone IM7, BioLegend
CD69-PE TexasRed, clone H1.2F3, BioLegend
IL-2-PE-Cy5, clone JES6-SH4, BioLegend
CD45.2-APC, clone 104, BioLegend
CD4-APC-Cy7, clone GK1.5, Thermo Fisher Scientific

### Statistical analysis

Comparisons between different groups of participants were evaluated using Student’s *t* tests, and comparisons between paired biopsies from the same participants were evaluated using paired *t* tests. Longitudinal repeated measures data was analyzed using mixed effects models with Greenhouse-Geisser correction and Tukey’s post-test. These analyses were conducted in Prism for Mac v8.4.3 (GraphPad). Flow cytometry data was visualized using dot plots and t-distributed stochastic neighbor embedding plots generated by FlowJo v10.7 (BD).

### Online supplementary material

Fig. S1 shows representative flow cytometry gating for human cells. Fig. S2 shows data from the mouse model demonstrating that tissue CD8 T cells retain function upon recurrent localized infection.

## Data Availability

Data are available in the article itself and its supplementary materials. Original flow cytometry data is available from the corresponding authors upon reasonable request.
